# Effect of Different Anthropometric Body Indexes on Radiation Exposure in Patients Undergoing Cardiac Catheterisation and Percutaneous Coronary Intervention

**DOI:** 10.3390/tomography8050189

**Published:** 2022-09-11

**Authors:** Youlin Koh, Sara Vogrin, Samer Noaman, Simon Lam, Raymond Pham, Andrew Clark, Leah Biffin, Laura B. Hanson, Jason E. Bloom, Dion Stub, Angela L. Brennan, Christopher Reid, Diem T. Dinh, Jeffrey Lefkovits, Nicholas Cox, William Chan

**Affiliations:** 1 Department of Cardiology, Western Health, Melbourne, VIC 3021, Australia; 2 Centre for Epidemiology and Biostatistics, University of Melbourne, Melbourne, VIC 3010, Australia; 3 Department of Cardiology, Alfred Health, Melbourne, VIC 3004, Australia; 4 Department of Radiology, Western Health, Melbourne, VIC 3021, Australia; 5 Baker Heart and Diabetes Institute, Melbourne, VIC 3004, Australia; 6 Department of Epidemiology and Preventative Medicine, Monash University, Melbourne, VIC 3004, Australia; 7 Centre of Cardiovascular Research and Education in Therapeutics, Monash University, Melbourne, VIC 3004, Australia; 8 Faculty of Medicine, Dentistry and Health Sciences, University of Melbourne, Melbourne, VIC 3010, Australia

**Keywords:** cardiac catheterisation, percutaneous coronary intervention, ionising radiation, body surface area

## Abstract

Background: Patient factors, such as sex and body mass index (BMI), are known to influence patient radiation exposure. Body surface area (BSA) and its association with patient radiation exposure has not been well studied. Methods and Results: We analysed height, weight, BMI and BSA in consecutive patients undergoing cardiac catheterisation and percutaneous coronary intervention (PCI) at a high-volume Australian centre between September 2016 and April 2020 to assess their association with dose–area product (DAP, Gycm^2^). The mean age of the cohort was 64.5 ± 12.3 years with males comprising 68.8% (*n* = 8100, 5124 diagnostic cardiac catheterisation cases and 2976 PCI cases). Median male BMI was 28.4 kg/m^2^ [IQR 25.2–32.1] versus 28.8 kg/m^2^ [24.7–33.7] for females, *p* = 0.01. Males had higher BSA (2.0 ± 0.2 m^2^) than females (1.78 ± 0.2 m^2^), *p* = 0.001. Each 0.4 m^2^ increase in BSA conferred a 1.32x fold change in DAP (95% CI 1.29–1.36, *p* ≤ 0.001). Each 5 kg/m^2^ increase in BMI was linked to a 1.13x DAP fold change (1.12–1.14, *p* ≤ 0.001). Male sex conferred a 1.23x DAP fold change (1.20–1.26, *p* ≤ 0.001). Multivariable modelling with BMI or BSA explained 14% of DAP variance (R^2^ 0.67 vs. 0.53 for both, *p* ≤ 0.001). Conclusions: BSA is an important anthropometric measure between the sexes and a key predictor of radiation dose and radiation exposure beyond sex, BMI, and weight.

## 1. Introduction

Radiation exposure to patients and staff in the catheterisation laboratory is an important procedural and safety metric, as tissue sensitivity to ionising radiation can result in stochastic and non-stochastic adverse effects. Stochastic injuries are related to cumulative radiation dose over time, potentially resulting in cancers affecting multiple organ systems [1,2]. Although modern radiation protection measures have reduced this risk, a modest increased incidence of solid tumours has been described in radiation workers [3]. Non-stochastic (or deterministic) effects are related to radiation dose above a certain threshold, leading to direct damage, such as skin burns or cataracts, which have been well-documented [4,5] but are beyond the scope of the current article.

In recent years, multiple studies have underscored specific patient and procedural factors that impact on radiation dose and, consequently, patient radiation exposure [6,7,8,9]. Several observational studies have assessed patient size as a predictor of radiation dose. Across a large (*n* = 20,000) cohort undergoing both diagnostic cardiac catheterisation and percutaneous coronary intervention (PCI), each 5.1 kg/m^2^ increase in body mass index (BMI) increased patient radiation exposure by 1.24-fold, after adjusting for multiple other procedural factors, such as lesion number and complexity [8]. Further dosimetry work by Madder et al. in a 1119-patient cohort [6] underscored the relationship between BMI and patient radiation dose but also showed that high patient BMI resulted in increased physician radiation dose [6]. However, alternative measures of patients, such as BSA, have not been examined to this extent in the setting of cardiac catheterisation, despite their established discriminative power in other fields of cardiology, such as echocardiography [10] and cardiac magnetic resonance imaging [11]. Thus, our study aims to evaluate different anthropometric measures in addition to other patient and procedural characteristics and their association with radiation dose and patient radiation exposure.

## 2. Materials and Methods

Consecutive patients at a high-volume Australian centre (Western Health, Melbourne, Victoria, Australia), from September 2016 and April 2020, undergoing cardiac catheterization and PCI, were included in the analysis. The study was approved by the Western Health Human Research and Ethics Committee. No informed consent was required. Anthropometric measures included height, weight, BMI, BSA by DuBois formula (0.20247 × height (m)^0.725^ × weight (kg)^0.425^). The DuBois formula was selected because of its longstanding use in the literature and high correlation with newer generation formulae, such as Gehan and George, and Mosteller [12]. Furthermore, direct BSA calculations show that the DuBois formula accuracy is less affected by body shape [13]. The selection of an appropriate BSA formula for the overweight population remains problematic [14].

Clinical characteristics and anthropomorphic measurements of the patient population were obtained from the Western Health cardiac catheterisation database. Patients who proceeded to PCI had additional periprocedural data recorded in the Victorian Cardiac Outcomes Registry (VCOR). VCOR is a state-wide clinical quality registry established to report on and improve the quality of care in cardiovascular disease in Victoria, Australia and has been previously described [15]. Data on all patients undergoing PCI in 32 public and private hospitals are collected as part of the VCOR PCI module [16]. The “PCI cohort” in this study included all patients who underwent PCI, comprising those who proceeded with ad hoc PCI (PCI following diagnostic cardiac catheterisation in the same procedure) and patients who had PCI only without preceding cardiac catheterisation.

Data on modes of clinical presentation, such as types of acute coronary syndrome (unstable angina, non-ST-elevation myocardial infarction [NSTEMI] and ST-elevation myocardial infarction [STEMI]) and stable angina, are captured by VCOR and are available for the PCI cohort.

Patients were categorised into tertiles of BSA with increments of 0.4 m^2^ based on work by Agarwal [17]. Multiple patient and procedural factors were also analysed together with different radiation outcome variables, which included fluoroscopy time, dose area product (DAP), air kerma and peak skin dose. As DAP is readily measurable and able to account for changes in tissue exposure secondary to angulation and tissue type [18], we utilised this as our outcome variable of interest in multivariate analyses. Furthermore, DAP incorporates the surface area of the patient irradiated and is more easily converted to effective dose, compared to air kerma [19,20]. Fluoroscopy time was not normalised to BSA group in our study.

Continuous variables are reported as mean ±SD or median (interquartile range). Categorical variables are reported as frequencies and percentages. Between-group univariate comparisons were performed using χ^2^ or Fisher’s exact tests for categorical variables, and ANOVA or Kruskal–Wallis for continuous variables. All tests were 2-tailed and assessed at the 5% significance level.

Multivariable linear regression was performed to identify significant predictors of DAP (defined as variables that were significant on multivariable testing). A stepwise backward regression was used for model development. Variables that met the pre-specified cut-off of *p* < 0.10 on univariable linear regression analysis were entered into multivariable analysis. As BMI and BSA were highly correlated with weight and height, only BMI or BSA were entered into each of the models. Increments of BMI of 5 kg/m^2^ and BSA of 0.4 m^2^ were selected to evaluate their relationship with DAP; these were chosen as they represent clinically meaningful categorical differences in previous studies [6,8,17,18]. Sidak correction for *p*-values was performed for multiple testing in the multivariable regression, yielding a cut-off of *p <* 0.0026 as the alpha value for each variable.

Additional analyses of model fit between a model without anthropometric measures and models containing one different anthropometric measure each were also performed. Model fit was evaluated by visual inspection of the residuals. Outcome (DAP) was transformed using natural logarithm to enable better model fit. Results are expressed as fold difference (exponentiated coefficients) with 95% confidence intervals. The proportion of variance in the outcome that can be explained by each anthropometric measure is presented as R^2^. All analyses were performed using StataCorp. 2019. Stata Statistical Software: Release 16. College Station, TX, USA: StataCorp LP. Data will be made available to researchers upon request by email correspondence.

## 3. Results

Baseline characteristics for the whole cohort (n = 8100) are summarised in Table 1. We analysed patients according to BSA tertiles: low < 1.8 m^2^, medium 1.8–2.0 m^2^, and high > 2.0 m^2^. We stratified BSA into these tertiles as these BSA increments have previously been used to detect differences in radiation exposure [17]. Patients in the low BSA group were older (mean age 70 for low vs. 66 for medium vs. 61 for high) and more likely to be female (60.2% vs. 26.8% vs. 13.0%), compared to medium and high BSA groups, all *p* < 0.001. Patients in the high BSA group were slightly more likely to have diabetes (high 36.3% vs. medium 33.7% vs. low 32.1%), *p* = 0.004 and a smoking history (27.9% vs. 26.9% vs. 23.1%), *p* < 0.001. Of 8100 patients, 2462 individuals proceeded with ad hoc PCI, and 514 individuals had PCI only as their procedure. Unfortunately, we were not able to obtain medication and laboratory parameters in all patients. However, the use of medications, such as hypoglycaemic agents and statins, are correlated with cardiovascular risk factors, such as diabetes and dyslipidaemia, which are included in our analysis.

Baseline characteristics of the PCI cohort (*n* = 2976, sum of ad hoc PCI and PCI only procedures) are summarised in Table 2. There was a similar preponderance of older females (mean age 68.2 vs. 63.4 vs. 60, *p* < 0.001) in the low BSA, compared to medium and high BSA groups. Among patients undergoing PCI, the prevalence of diabetes (low 26.5% vs. medium 25.5% vs. high 28.2%), and a smoking history (31.7% vs. 32.1% vs. 27.3%) was not significantly different between BSA groups.

In the whole cohort, (Table 1), patients in the low BSA group were significantly more likely to undergo femoral access (low 27.9% vs. medium 24.6% vs. high 21.1%). Females are known to have smaller radial artery diameters than men [21,22], and this might have favoured a femoral approach to avoid radial artery spasm, improve procedural efficiency and reduce the need for vascular access site cross-over.

Among patients in the PCI cohort, those in the low and medium BSA groups were more likely to undergo PCI for unstable angina (low 3.6% vs. medium 3.0% vs. high 5.8%) and NSTEMI (low 33.9% vs. medium 30.7% vs. high 28.0%), all *p* = 0.001. Conversely, there were more STEMI presentations in the high and medium BSA groups (high 28.5% vs. medium 28.6% vs. low 25.0%). Ad hoc PCI occurred more frequently in the low and medium BSA groups (low 82.4% vs. medium 84.2% vs. high 81.6%), *p* < 0.001. Complex PCI (defined as those undergoing left main PCI, rotablation, chronic total occlusion PCI or intravascular ultrasound [IVUS] imaging) case numbers were similar between BSA groups (low 7.6% vs. medium 6.0% vs. high 5.7%), *p* = 0.66. There was also a greater use of the femoral approach in low BSA patients (low 31.4% vs. medium 23.7% vs. high 21.8%), *p* < 0.001.

Previous studies have noted sex differences in radiation exposure, with a higher dose delivered to male patients [23]. However, measures of patient size underlying this difference have not been explored. We, therefore, analysed multiple anthropometric measurements and their associated sex differences. Median height and weight were both higher in males (173 cm [IQR168–178] vs. 160 cm [IQR 155–165], *p* ≤ 0.001 and 85 kg [IQR 75–98] vs. 74 kg [IQR 63–86] for males and females, respectively). Median BMI was lower for males, compared to females (28.4 kg/m^2^ [25.2–32.1] vs. 28.8 kg/m^2^ [24.7–33.7]), *p* = 0.01. However, BSA was higher in males, compared with females (mean 2.0 ± 0.2 m^2^ vs. 1.78 ± 0.2 m^2^, *p* = 0.001).

**Figure 1:**
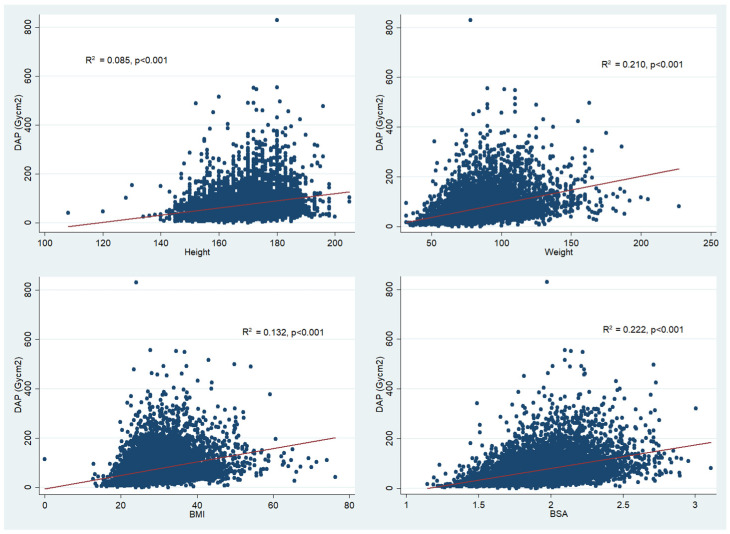
Correlation of different anthropometric indices with dose area product (DAP). R^2^ = correlation coefficient, and represents proportion of variance in DAP explained by each variable in linear regression model. BMI, body mass index; DAP, dose area product.

We systematically assessed the correlation of various anthropometric indices with DAP in the whole cohort (Figure 1). All parameters studied were significantly and weakly correlated; however, BSA accounted for the greatest proportion of variance in DAP (R^2^ = 0.222, *p* < 0.001). The correlation of weight with DAP was similar (R^2^ = 0.210, *p* < 0.001). The relationships between BMI and DAP (R^2^ = 0.132, *p* < 0.001), and height and DAP (R^2^ = 0.085, *p* < 0.001) were less robust. As shown in Table 3, fluoroscopy time was higher in the low and medium BSA groups, compared to the high BSA group (3.5 min vs. 3.6 min vs. 3.2 min/m^2^. DAP rose steeply between the low, medium and high BSA groups (median 36.7 vs. 56.2 vs. 79.9 Gycm^2^), all *p* < 0.001. Similarly, increases in air kerma and peak skin dose between low, medium and high BSA tertiles were observed (506 vs. 758 vs. 1070 mGy, and 200 vs. 320 vs. 440 mGy), all *p* < 0.001.

Among those in the PCI cohort, as shown in Table 4, DAP, air kerma and peak skin dose all markedly rose with increasing BSA (low 63.8 vs. medium 82.4 vs. high 119 Gycm^2^; low 991 vs. medium 1260 vs. high 1800 mGy; and low 520 vs. medium 680 vs. high 940 mGy), all *p* < 0.001.

**Figure 2:**
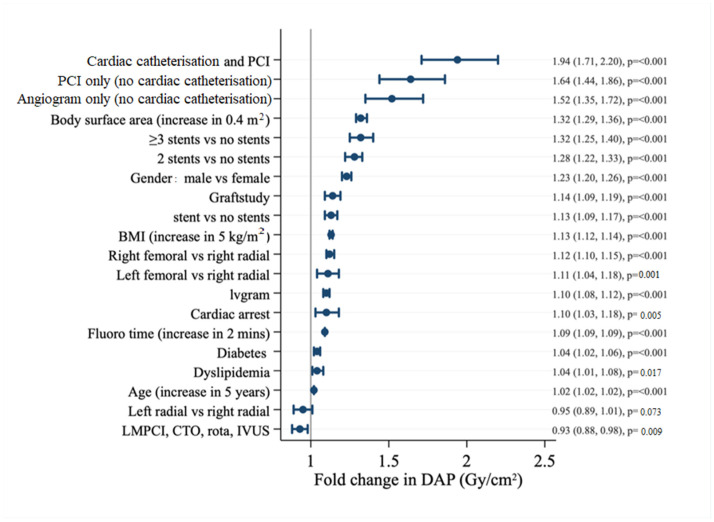
Multivariate analysis of whole cohort. BMI, body mass index; CTO, chronic total occlusion; DAP, dose area product; IVUS, intravascular ultrasound; LMPCI, left main percutaneous coronary intervention; PCI, percutaneous coronary intervention; rota, rotablation.

Each 0.4 m^2^ increase in BSA was associated with a 1.32 (95% confidence interval, [CI] 1.29–1.36, *p* ≤ 0.001) fold change in DAP (Figure 2). Each 5 kg/m^2^ increase in BMI conferred a 1.13x fold change (95% CI 1.12–1.14, *p* ≤ 0.001). Male sex was associated with a greater increase in DAP (1.23, [CI 1.22–1.33], *p* ≤ 0.001). The association between age (per 5-year increase) and DAP was modest (1.02, [CI 1.02–1.02], *p* ≤ 0.001).

Femoral access was associated with an increase in DAP (1.12 [CI 1.10–1.15], *p* ≤ 0.001 for right femoral and 1.11 [CI 1.04–1.18], *p* = 0.001 for left femoral, respectively). Each two-minute increase in fluoroscopy time conferred a 1.09-fold change in DAP [CI 1.09–1.09, *p* ≤ 0.001). The lower DAP observed with complex PCI, such as left main and chronic total occlusion PCI and IVUS use, was likely due to a few reasons. Firstly, the smaller number of patients undergoing these procedures likely accounted for this observation (Table 2); secondly, most of these patients would already have had prior coronary angiography to define the complexity of the disease, and, therefore, the PCI procedure would have been planned ahead of time and focussed on the PCI aspect only; and thirdly, IVUS use during these procedures would likely have reduced the need for additional fluoroscopy or cineangiography.

**Figure 3:**
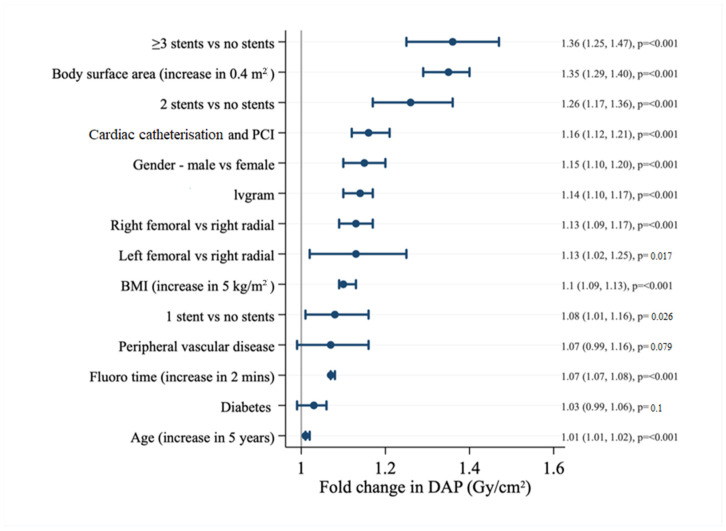
Multivariate analysis of PCI cohort. BMI, body mass index; DAP, dose area product; IVUS, intravascular ultrasound; PCI, percutaneous coronary intervention.

The relationship between increasing BSA and DAP was similar in magnitude for the PCI cohort, as observed for the whole cohort (fold change 1.35 [CI 1.29–1.40], *p* ≤ 0.001) (Figure 3). Likewise, increasing BMI was associated with a 1.1x increase in DAP [CI 1.09–1.13, *p* ≤ 0.001]. Sex differences in DAP persisted (fold change 1.15 for males, compared to females, [CI 1.10–1.20], *p* ≤ 0.001).

Ad hoc PCI was associated with an increase in DAP (fold change 1.9 [CI 1.71–2.20] versus 1.52 [CI 1.35–1.72] for cardiac catheterization); PCI only was 1.64 ([CI 1.44–1.86], *p* ≤ 0.001). Incremental increases in DAP were observed with multiple stent implantation, (1.08 [CI 1.01–1.16], *p* = 0.026) for single stent; 1.26 [CI 1.17–1.36], *p* ≤ 0.001 for two stents; and 1.36 [CI 1.25–1.47], *p* ≤ 0.001 for three or more stents, respectively).

The multivariable model (Figure 2) without any anthropometric measures has an R^2^ of 0.53, with the included clinical and procedural factors explaining approximately 53% of the observed variance in DAP. Adding height to the model (Table 5) only increased R^2^ to 0.54, *p* = 1.000. In contrast, adding weight significantly improved DAP discrimination (R^2^ = 0.69, *p* ≤ 0.001). Both BMI and BSA accounted for a similar amount of DAP variance, (R^2^ = 0.67 for BMI and R^2^ = 0.67 for BSA, respectively, all *p* ≤ 0.001).

## 4. Discussion

In this large study of 8100 consecutive patients undergoing cardiac catheterisation and PCI, all anthropometric indices had significant association with DAP but BSA demonstrated the highest association with DAP. However, the difference in BSA magnitude between sexes was greater than that of BMI values between the sexes. These findings underscore the key anthropometric differences between the sexes and their effect on consequent patient radiation exposure. Previous work has shown that despite an increase in radiation exposure in male patients, commonly used patient size measurements, such as BMI, have not effectively captured this difference with very similar median values obtained between the sexes [23]. Our findings replicate, in a large multicultural Australian population, the finding that BSA is associated with increased patient radiation exposure, similar to international experience in the United States [24] and Europe [25]. Our study also underscores BSA as a potential reason for sex-based differences in patient size and radiation exposure.

Despite our findings, most studies of cardiac catheterisation have focused on BMI as a measure of adult patient size in evaluating its relationship with radiation exposure [6,7,8,18]. Indeed, it appears that BSA is mostly used in the paediatric population for the purpose of indexing radiation dose to patient size [26,27,28]. Yet, the few adult studies that have utilised BSA for anthropometric measurement also demonstrate a dose–response relationship between BSA and radiation exposure, usually quantified by DAP [17,24]. For example, in a study by Agarwal et al. [17], increasing BSA was associated with increasing air kerma rate (mGy/s, indexed to fluoroscopy time). At a BSA of 1.6 m^2^, the air kerma rate was approximately 0.8 mGy/s, compared to 1.2 mGy/s at a BSA of 2.0 m^2^, and 2 mGy/s at a BSA of 2.4 m^2^. A study by Memon et al. [24] showed that use of a low-dose X-ray system was associated with a significant reduction in DAP, with the greatest reduction seen in the highest BSA quartile (>2.2 m^2^) from a mean DAP of 159,224 mGycm^2^ to 69,386 mGycm^2^. In a radial access cohort, Mantis et al. [25] also demonstrated that increasing BMI, BSA, male sex and older age were associated with increasing DAP and air kerma. In our study, we additionally performed multiple multivariable models evaluating the effect of individual anthropometric indexes on patient radiation exposure, thereby providing a comparison between different anthropometric measures.

DAP is the product of the surface area of the patient that is exposed to radiation (at the skin entry) and the radiation dose delivered at this surface and is usually measured in an ionisation chamber at the level of the X-ray tube [29]. The method by which DAP is increased in high BSA or overweight patients is due to the increase in X-ray dose required to increase image quality as the rays traverse larger amounts of tissue [18,19].

Although all anthropometric measurements varied significantly between the sexes, there was a larger numerical difference observed in weight and BSA in our study cohort. Previous studies have highlighted that men receive higher radiation doses during cardiac catheterisation, compared to women, despite both groups having similar median BMIs [23,30]. In these studies, males had a higher median weight, but BSA notably was not examined. Our results show that sex-based body size differences are key patient factors that impact DAP even more so than procedural factors, such as vascular access or procedural complexity (unless it involved >3 stents). Weight is also a good discriminator of patient size and demonstrated marginal improved performance to BSA in our multivariate model for DAP. However, while simple to measure, weight cannot be easily modified in the cardiac catheterisation laboratory, whereas the amount of BSA exposed could be a modifiable patient factor that alters radiation exposure. BSA has also been postulated to be an accurate indicator of metabolic mass [31], and likely explains our finding of BSA discriminating well between the sexes.

Simple radiation practices, such as beam collimation [32], using shallow angulation, different rotating projections where possible and real-time patient radiation exposure feedback [33], could all engender meaningful reduction in radiation dose and DAP in high BSA patients. Our catheterisation laboratory has also implemented a default low frame rate, with protocol similar to that of Chon and colleagues [34]; further gains may be made in considering alternative software, such as AlluraClarity [24].

As the amount of BSA exposed is potentially modifiable, the development of non-lead materials to reduce radiation scatter in patients with a large body size is an important area of future research. Existing studies, such as that by Musallam et al., examined the utility of patient pelvic lead shields [35], showing that patients in the shielded group received double the standard effective doses in ad hoc PCI [36], with the hypothesis that this was related to increased backscatter from the lead shield. Radiation scatter is increased in obese patients, leading to reduced image quality and the increased use of scatter grids, which then require increased X-ray exposure in order to maintain image quality [37]. Other studies utilising bismuth-based shielding have produced similar findings, with recorded non-significant increases in patient radiation dose and decreased operator dose [38,39].

In our study, ad hoc PCI was associated with the highest increase in DAP by approximately 1.9 times, compared to diagnostic cardiac catheterisation, which was 1.5 times. However, PCI timing is often affected by multiple factors, including the desire to limit potential complications with repeated procedures, in which case the ad hoc approach would be more beneficial, for example, in patients with ongoing ischaemia [40]. Another important consideration in mitigating radiation dose is limiting the number of stents deployed during PCI [8]. In our study, the greatest effect on DAP was observed between the first and second stent with more than three stents implanted associated with significant increase in DAP.

Our study findings need to be interpreted in context of several limitations. The retrospective design of our study limits conclusions that can be drawn regarding any causal relationships. Secondly, there are confounders that could have affected DAP that were not captured or adjusted for, such as day-to-day and between-operator variations in radioprotective behaviours. Thirdly, patient radiation dose was not measured with dosimetry, which would have provided more direct dose quantification. We also did not record other anthropometric measures, such as waist size, which could potentially be an important factor in the evaluation of patient radiation exposure, given its relevance to body shape. However, waist size has been shown to be highly correlated with other anthropometric measures, such as BMI [41], which was reported and analysed in our study. Finally, our dataset did not include medications and biochemical laboratory parameters so their potential association with patient radiation exposure was not able to be assessed.

## 5. Conclusions

BSA is an important anthropometric measure between the sexes and a key predictor of radiation dose and radiation exposure beyond sex, BMI and weight. Although weight and sex are difficult to modify, measures to reduce BSA during cardiac catheterisation, for example, use of non-lead shielding materials, could reduce DAP, and it warrants further research. Simple radiation mitigation practices, the use of novel shielding equipment, as well as careful pre- and intra-procedural planning during cardiac catheterisation and PCI are critical in minimising radiation dose and exposure in patients with high BSA.

## Figures and Tables

**Table 1:** Baseline characteristics for whole cohort, analysed by body surface area categories. Both patient and procedural characteristics are described.

Factor	BSA < 1.8 m^2^	BSA 1.8–2.0 m^2^	BSA > 2.0 m^2^	*p*-Value
No. of patients	2322	3079	2699	
Age, mean (SD)	70 (12.2)	66 (12.1)	61 (12.0)	<0.001
Female	1397 (60.2%)	724 (26.8%)	400 (13.0%)	<0.001
Height, mean (SD)	155 (7.3)	170 (7.2)	176 (7.4)	<0.001
Weight, median (IQR)	64 (59–70)	80 (75–84)	100 (91–110)	<0.001
Body Mass Index (BMI), median (IQR)	24.6 (22.2–27.5)	27.6 (25.1–30.8)	32.4 (29.3–36.8)	<0.001
Peripheral vascular disease	76 (3.3%)	104 (3.9%)	85 (2.8%)	0.067
Diabetes	745 (32.1%)	909 (33.7%)	1117 (36.3%)	0.004
Dyslipidaemia	114 (4.9%)	203 (7.5%)	219 (7.1%)	<0.001
Hypertension	594 (25.6%)	606 (22.5%)	704 (22.9%)	0.02
Ever smoker	537 (23.1%)	726 (26.9%)	859 (27.9%)	<0.001
Cardiac arrest	17 (0.73%)	49 (1.82%)	64 (2.08%)	<0.001
Cardiac catheterisation only (no PCI)	1463 (63.0%)	1478 (54.8%)	1710 (55.5%)	<0.001
PCI only (no cardiac catheterisation)	128 (5.5%)	167 (6.2%)	219 (7.1%)	0.137
Ad hoc PCI	600 (25.8%)	890 (33.0%)	972 (31.6%)	<0.001
Access site				<0.001
Right radial	1615 (69.6%)	1970 (73%)	2360 (76.7%)	
Left radial	51 (2.2%)	54 (2.0%)	62 (2.0%)	
Right Femoral	597 (25.7%)	609 (22.6%)	597 (19.4%)	
Left Femoral	52 (2.2%)	55 (2.0%)	52 (1.7%)	
Other	7 (0.30%)	11 (0.41%)	7 (0.23%)	
Left ventriculography	1659 (71.5%)	1924 (71.3%)	2242 (72.8%)	0.364

* Abbreviations: BMI, body mass index; BSA, body surface area; PCI, percutaneous coronary intervention.

**Table 2:** Baseline characteristics for whole cohort, analysed by body surface area categories. Both patient and procedural characteristics are described.

Factor	BSA < 1.8 m^2^	BSA 1.8–2.0 m^2^	BSA > 2.0 m^2^	*p*-Value
No. of patients	728	1057	1191	
Age, mean (SD)	68.2 (12.5)	63.4 (12.0)	60 (11.3)	0.009
Female	363 (49.9%)	199 (18.8%)	107 (9.0%)	<0.001
Height, mean (SD)	161 (6.9%)	170 (6.8%)	176 (7.0%)	<0.001
Weight, median (IQR)	65 (60–69)	80 (75–83)	98 (90–110)	0.0001
Body Mass Index (BMI), median (IQR)	24.2 (22.1–27.0)	27.0 (25.0–30.1)	31.7 (28.9–35.9)	0.0001
Peripheral vascular disease	26 (3.6%)	42 (4.0%)	35 (2.9%)	0.400
Diabetes	205 (28.2%)	270 (25.5%)	316 (26.5%)	0.465
Dyslipidaemia	42 (5.8%)	96 (9.1%)	103 (8.7%)	0.023
Hypertension	185 (25.4%)	213 (20.2%)	242 (20.3%)	0.014
Ever smoker	199 (27.3%)	339 (32.1%)	377 (31.7%)	0.069
Cardiac arrest	17 (2.3%)	45 (4.3%)	60 (5.0%)	0.01
Acute coronary syndrome type				
Unstable angina	26 (3.6%)	32 (3.0%)	69 (5.8%)	0.001
NSTEMI	247 (33.9%)	324 (30.7%)	333 (28.0%)	
STEMI	182 (25.0%)	302 (28.6%)	339 (28.5%)	
Elective	273 (37.5%)	399 (37.8%)	450 (37.8%)	
Ad hoc PCI	600 (82.4%)	890 (84.2%)	972 (81.6%)	0.260
Complex PCI	55 (7.6%)	63 (6.0%)	68 (5.7%)	0.239
Left main PCI	11 (0.15%)	21 (0.20%)	13 (0.11%)	0.221
CTO	30 (4.12%)	31 (1.15%)	49 (1.59%)	0.261
Rotablation	11 (0.47%)	10 (0.37%)	7 (0.23%)	0.127
IVUS	15 (0.65%)	26 (0.96%)	22 (0.71%)	0.598
Graft study	20 (2.8%)	30 (2.8%)	23 (1.9%)	0.321
Access site				0.001
Right radial	486 (66.8%)	792 (75.9%)	908 (76.2%)	
Left radial	10 (1.4%)	11 (1.0%)	18 (1.5%)	
Right Femoral	212 (29.1%)	228 (21.6%)	235 (19.7%)	
Left Femoral	17 (2.3%)	22 (2.1%)	25 (2.1%)	
Other	3 (0.41%)	4 (0.38%)	5 (0.42%)	
Left ventriculography	451 (62%)	697 (65.9%)	749 (62.9%)	0.165
Number of stents				
0	32 (4.4%)	46 (4.4%)	57 (4.8%)	0.814
1	430 (59.7%)	627 (59.3%)	732 (61.5%)	
2	191 (26.2%)	269 (25.5%)	277 (23.3%)	
≥3	75 (10.3%)	115 (10.9%)	125 (10.5%)	

Abbreviations: BMI, body mass index; BSA, body surface area; CTO, chronic total occlusion; IVUS, intravascular ultrasound; NSTEMI, non-ST-elevation myocardial infarction; PCI, percutaneous coronary intervention; STEMI, ST-elevation myocardial infarction.

**Table 3:** Radiation measures for whole cohort, including indexed fluoroscopy time, dose–area product, air kerma and peak skin dose.

Factor	BSA < 1.8 m^2^	BSA 1.8–2.0 m^2^	BSA > 2.0 m^2^	*p*-Value
Fluoroscopy Time Indexed to BSA (min/m^2^), median (IQR)	3.5 (1.9–6.3)	3.6 (1.9–5.9)	3.2 (1.7–5.5)	0.0001
DAP Gycm^2^, median (IQR)	36.7 (23.6–59.2)	56.2 (37.1–86.0)	79.9 (52.3–123)	<0.001
Air Kerma mGy, median (IQR)	506 (323–840)	758 (497–1240)	1070 (689–1740)	<0.001
Peak Skin Dose mGy, median (IQR)	200 (120–410)	320 (180–640)	440 (250–890)	<0.001

Abbreviations: BSA, body surface area; DAP, dose area product.

**Table 4:** Radiation measures for PCI cohort, including indexed fluoroscopy time, dose–area product, air kerma and peak skin dose.

Factor	BSA < 1.8 m^2^	BSA 1.8–2.0 m^2^	BSA > 2.0 m^2^	*p*-Value
Fluoroscopy Time Indexed to BSA (min/m^2^), median (IQR)	6.8 (4.7–9.8)	5.7 (4.0–8.3)	5.3 (3.7–8.0)	0.0001
DAP Gycm^2^, median (IQR)	63.8 (44.6–94.1)	82.4 (60.7–120)	119 (85.0–169)	<0.001
Air Kerma mGy, median (IQR)	91 (673–1490)	1260 (880–1820)	1800 (1270–2560)	<0.001
Peak Skin Dose mGy, median (IQR)	520 (350–800)	680 (460–970)	940 (650–1380)	<0.001

* Abbreviations: BSA, body surface area; DAP, dose area product; PCI, percutaneous coronary intervention.

**Table 5:** Comparison of model fit with addition of different anthropometric variables. R^2^ describes the proportion of the variance in DAP that can be explained by the multivariable model.

	R^2^	*p*-Value
Model without anthropometric measures	0.531	
Model + height	0.544	1.000
Model + weight	0.686	≤0.001
Model + BMI	0.673	≤0.001
Model + BSA	0.672	≤0.001

* Abbreviations: BMI, body mass index; BSA, body surface area.

## Data Availability

The data presented in this study are available on request from the corresponding author. The data are not publicly available in order to maintain patient confidentiality.
